# The Effect of Hypochlorous Acid Disinfectant on the Reproduction of Details and Surface Hardness of Type III Dental Stone

**DOI:** 10.7759/cureus.32061

**Published:** 2022-11-30

**Authors:** Zaid M Jasim, Shorouq M Abass

**Affiliations:** 1 Department of Prosthodontics, College of Dentistry, University of Baghdad, Baghdad, IRQ

**Keywords:** dentistry, surface hardness, reproduction of details, dental stone, hypochlorous acid

## Abstract

Introduction

Gypsum products have been used for many years in dentistry. They are used to make casts that are used in different dental laboratory procedures. It is considered a source of contamination because it comes in contact with blood and saliva that are found on dental impressions. Because it is difficult to make sure that every impression brought to the dental laboratory has been cleaned and because cleaning impressions is a complicated process that can lead to problems because of the way impression materials are made, cleaning the cast has become a key part of preventing infections. For this study, we used two ways (immersion and spray) to apply a hypochlorous acid (HOCl) disinfectant solution to type III dental stone to see how they affected the stone's surface hardness and its ability to show surface details accurately.

Materials and methods

A total of 100 samples of type III dental stone, 50 samples for each test, which include the reproduction of detail test and surface hardness test, were prepared and randomly divided into five groups, 10 samples for each test group, which include group A (control), group B (immersion in 200 ppm HOCl disinfectant for five minutes), group C (spraying with 200 ppm HOCl disinfectant for five minutes), group D (immersion in 0.6% sodium hypochlorite (NaOCl) disinfectant for five minutes), and group E (spraying with 0.6% NaOCl disinfectant for five minutes).

Results

The results showed that all the samples had met the reproduction of detail test requirement after disinfection with HOCl, while a significant reduction in the surface hardness of type III stone samples was also shown.

Conclusion

The less undesirable effects of HOCl disinfectant solution on the surface hardness of type III dental stone when compared to sodium hypochlorite, as well as the absence of adverse effects on detail reproduction, made the HOCl disinfectant solution a good choice for dentists and dental laboratory personnel for cast disinfection and contamination control.

## Introduction

Gypsum and its products have been used for many years in different fields. In dentistry, it is mostly used to make casts and for various dental laboratory procedures. Restorative dentistry poses a particularly high risk for cross-contamination with stone casts because of the many routes by which infectious pathogens may be transferred from the patient's saliva to the castings. Therefore, following each clinical and laboratory procedure, these casts should be disinfected [1]. The potential for getting and/or transferring severe infectious diseases is the most serious threat that a dental professional faces. Equipment supplies, tools, impressions, and casts have been shown to be a possible source of microbial infection since they may facilitate the transmission of disease via blood and saliva. Therefore, extra care must be given when developing, fabricating, and handling prosthodontic restorations [2].

From a dental laboratory point of view, it may be more efficient to remove any obvious contaminants, such as blood and saliva, by washing away the impressions before the stone cast pouring and thereafter sterilizing the cast [3]. If you are going to use dental impressions or gypsum casts, you should clean them beforehand, as recommended by the American Dental Association (ADA) and the Centers for Disease Control and Prevention (CDC). Cast disinfection has become a key step in creating clean models and developing a cross-contamination control process because impression disinfection is challenging and associated with several problems [4]. Disinfectant solutions must kill microbes well without hurting the cast or die's physical properties, such as gypsum's ability to stay the same size [5].

A study done by Michael et al. stated that after 24 hours of disinfection by both spray disinfection and immersion disinfection processes with 0.5% sodium hypochlorite (NaOCl), the stone specimens had an adverse effect on their mechanical properties when compared with the control group specimens [6], while another study [7] suggested that type III and IV dental stones had dramatically reduced mechanical characteristics after being chemically disinfected with 1% Virkon in slurry, 0.525% sodium hypochlorite in slurry, and a separate slurry solution [3]. Non-toxic, corrosive-free, efficient in a variety of formulations, and inexpensive are all desirable qualities in a disinfectant or sanitizer. It is possible that hypochlorous acid (HOCl) is the most effective disinfectant [8]. Hypochlorous acid (HOCl), a non toxic antimicrobial agent, is utilized to prevent illness, decrease inflammation, and hasten the healing of wounds with fewer adverse side effects. In vitro studies have also shown that HOCl has a profound effect on oral microbes such as *Streptococcus mutans*, *Streptococcus sanguinis*, *Porphyromonas gingivalis*, *Aggregatibacter actinomycetemcomitans*, *Campylobacter rectus*, and enteric rods that live in the dental biofilm of the teeth and dental implants [8]. In this study, the hypochlorous acid disinfection solution was used in two different ways (immersion and spray) to see how they affected the stone's surface, how well details showed up, and how hard the surface was.

## Materials and methods

Sample preparation

All of the stone samples adhered to ADA specification number 25 for gypsum products in terms of the mixing process used to prepare them. Using an electronic balance and a measuring cylinder, we combined all of the samples to the manufacturer's recommended water/powder ratio. Type III dental stone powder (Elite Model, Zhermack, Badia Polesine, Italy) weighed 100 g per 30 mL of water. The standard mix was made by adding, over a period of 10 seconds, the dry powder to the recommended amount of distilled water in a clean rubber bowl. The mixture was allowed to mix for 30 seconds to a smooth consistency. After mixing, the mixture was put into a rubber ring measuring 15 mm in height and 30 mm in diameter, which was utilized to create stone samples. Pouring the combined dental stone mixture through a vibrator to eliminate air bubbles and reduce porosity is how the American Dental Association recommends it be done. In order to generate samples with smooth, parallel surfaces, glass slabs were put on the farthest edges of the rubber ring. After one hour of mixing, each stone sample was carefully removed from the rubber ring and left at a constant temperature of 23°C for 24 hours with a relative humidity of 50%.

Hypochlorous acid disinfectant solution preparation

On-site production of hypochlorous acid disinfection solutions is possible using electrolysis of non-iodized salt and water. Using the Eco One (EcoloxTech, West Palm Beach, FL) system, one may make HOCl on-site by adding 2 g of non-iodized salt (such as kosher salt) and one teaspoon of white vinegar (5%) to a 1.5 L container containing 1 L of water. Vinegar may be replaced with acetic acid to keep the pH of 4.5. HOCl is the most abundant free chlorine molecule at a pH of 4.5. HOCl solutions need to be between 3.5 and 5 on the pH scale to keep their antibacterial properties, kill the most bacteria, and make the least amount of unwanted by-products. A pilot study was done to choose the most appropriate concentration of acetic acid in order to make the pH equal to 4.5. The pilot study showed that adding 525 µL of 99.9% concentrated acetic acid (Solvochem, Rotterdam, the Netherlands) to 1 L of distilled water with 2 g of non-iodized salt and pressing on setting 3 of the device two times (each time for eight minutes to complete the cycle) made the pH of the resultant HOCl equal to 4.5 [9-11].

Sodium hypochlorite disinfectant solution preparation

The solution of NaOCl (Fas, Baghdad, Iraq) with a concentration of 6% was diluted to 0.6% by the use of bleach solution of hypochlorite and diluted water with a ratio of 1:10 (10 parts of water to one part of bleach) as stated by the ADA recommendation for disinfection [11].

Sampling groups of the main study

A total of 100 samples of type III dental stone (50 samples for each test) were prepared and randomly divided into five groups, with 10 samples for each test group: test group A, a control group with no disinfectant treatment (rinsed with distilled water); test group B, immersion in 200 ppm HOCl disinfectant for five minutes; test group C, spray with 200 ppm HOCl disinfectant for five minutes; test group D, immersion in 0.6% NaOCl disinfectant for five minutes; and test group E, spray with 0.6% NaOCl disinfectant for five minutes.

Disinfection of stone specimens by the spraying method

The stone model specimen was positioned in the middle of a deep container and disinfected by spraying. The spraying is done by a sprayer held at a constant distance and at a right angle to the surface of the stone sample in order to standardize the spraying procedure. Each sample was sprayed until the surface of the stone was saturated, at which point the liquid spray no longer penetrated the stone and left a liquid residue visible on the stone's surface. The stone model was let stand for five minutes after spraying was complete. The samples were removed from the container, rinsed with distilled water, and left to dry for one hour at room temperature (23°C) and relative humidity (50%) before being stored in a desiccator until testing. A pair of tweezers was used to place and remove stone samples from the disinfecting solution [6].

Disinfection of stone samples by immersion method

The stone samples were soaked in a soaking solution for five minutes at room temperature in a reasonably sized container filled with 1 L of the prepared (0.6%) sodium hypochlorite and 1 L of the prepared (200 ppm) hypochlorous acid. After being withdrawn from the solution, the samples were washed with distilled water, dried for an hour at ambient temperature (23°C) and relative humidity (50%), and then stored in a desiccator until testing was ready to begin. A pair of tweezers was used to pick up the stone specimens into and from the disinfectant solution.

Reproduction of detail test

The samples' ability to reproduce details was evaluated according to ADA specification number 25. According to ADA specification number 25, a test block was produced with seven 60° angle grooves at specified widths of 0.025 mm, 0.05 mm, 0.075 mm, 0.1 mm, 0.15 mm, 0.2 mm, and 0.3 mm, and a crossline running across these furrows. Stone samples were poured over the testing block using an elastic ring 30 mm wide and 15 mm high. It was determined by positioning the test block under the elastic ring so that the point of intersection between the crossline and the line 0.05 mm in width was at the center of the ring. The block and the ring vibrated at the same time. The ring was filled with the premixed mixture. After waiting for 60 minutes, the ring and stone samples were removed from the test block and set aside for evaluation. The detail reproduction was considered to be satisfactory if the 0.05 mm wide groove was clear and continuous for the full length of the ring diameter. The line reproduction quality was evaluated by visual observation (without magnification) under low-angle illumination. As recommended by the WHO [12], 10 dentists looked at and evaluated the samples twice, with 10-14 days between the first and second readings. The evaluations were carried out in an arbitrary order, and the examiners had no idea what kind of examples they were judging. A scale from "I" to "IV" was used to assign a score to each case: Score I, the line is straight and continuous across the entire ring's width; score II: the line is clear and consistent across more than half of the ring's width; score III: the line is continuous and clear across only a fraction of the ring's width; score IV: the line fails to be repeated along the ring's width.

Surface hardness test

**Figure 1:**
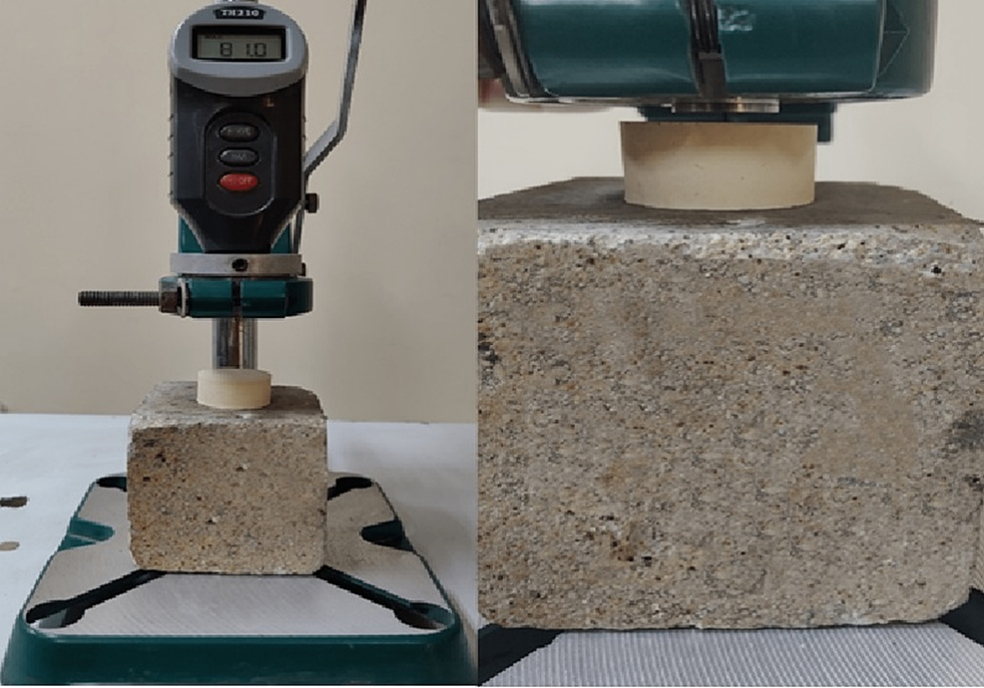
Surface hardness test with Shore D hardness tester

All the specimens were tested with a Shore D hardness tester (TIME Group Inc., Beijing, China) [13]. The pointed indenter penetrated the surface of the sample (Figure 1), and the result of the hardness was noted approximately after five seconds on the screen of the device.

The results are presented as an average of five measurements for each sample [14].

## Results

The results of the effect of using 200 ppm hypochlorous acid disinfectant solution with immersion and spray methods on the reproduction of details and surface hardness of type III dental stone were as follows.

Reproduction of details

The scores that were recorded by each examiner for each of the test groups were found to be in total agreement with each other. All of the test groups' samples passed the test by making a complete, well-defined groove that was 0.050 mm wide. This meant that they met the ADA specification.

Surface hardness

The change in the surface hardness as a result of the disinfection of type III dental stone samples by hypochlorous acid disinfection solution with immersion and spray methods was recorded in terms of a change in the Shore D hardness number after 24 hours. The mean values for the test groups were 82.41, 79.03, 80.3, 76.98, and 80.18 after 24 hours for groups A, B, C, D, and E, respectively. The highest was for group A (control) and then C, E, and B, and the least was for group D (immersion in NaOCl). The statistics of Shore D hardness test results utilizing one-way analysis of variance (ANOVA) for the comparison of mean values of control and test groups are listed in Table 1.

**Table 1:** Statistics of Shore D hardness test SD: standard deviation; HOCl: hypochlorous acid; NaOCl: sodium hypochlorite

Groups	N	Mean	SD	F-value	P-value
Group A (control)	10	82.41	1.4	29.932	0.001
Group B (HOCl immersion)	10	79.03	0.9		
Group C (HOCl spray)	10	80.3	0.7		
Group D (NaOCl immersion)	10	76.98	1.5		
Group E (NaOCl spray)	10	80.18	1.1		

There was a statistically significant difference between the groups (P<0.001). The results of the Shore D hardness test showed a very significant difference (P=0.001) between groups B, C, D, and E when compared with the control (group A) (less than control) after a post hoc least significant difference (LSD) test was conducted. The comparison between groups B and D for the immersion approach and groups C and E for the spraying method showed a very significant difference (P=0.001) between groups B and D but no significant difference (P=0.822) between groups C and E (Table 2).

**Table 2:** Multiple comparisons by a post hoc test (LSD) for the data of Shore D hardness test HS: highly significant; NS: not significant; LSD: least significant difference

Groups compared	Mean difference	P-value	Significance
A and B	3.38	0.001	HS
A and C	2.11	0.001	HS
A and D	5.43	0.001	HS
A and E	2.23	0.001	HS
D and B	-2.05	0.001	HS
E and C	-0.12	0.822	NS

## Discussion

Every dental professional must do their work in a way that prevents cross-contamination and the transmission of infection. Due to the COVID-19 pandemic and the rise of communicable diseases such as HIV/AIDS, hepatitis B, and hepatitis C, it is very important to make sure that dental workers and patients don't get sick from each other by taking the right steps to disinfect [15]. Microorganisms from the patient's saliva and blood may readily transfer to stone castings made from oral impressions. Recent research shows that oral bacteria can live for up to seven days in gypsum that has hardened [16]. Additionally, there are a few issues connected with the chemical cleaning of impressions, such as the fact that it is time-consuming and costly and requires a newly made solution at a dental office. Contamination by microorganisms may still exist after cleaning efforts [17]. Sterilization methods are now widely used in healthcare systems, but all means of sterilization are not suitable for dental stone casts due to the adverse damage expected. To minimize the spread of illness, the American Dental Association and the Centers for Disease Control and Prevention have recommended that dental impressions and gypsum casts be sterilized before use [17].

Dental stone casts should be disinfected by either being submerged in a disinfectant solution (hypochlorite or iodophor) or being sprayed with the solution. These procedures are suggested by both the CDC and the American Dental Association [17]. Some researchers have looked into the effects of incorporating different sanitizers on the physical properties, dimensional stability, and surface porosity of the dental stone. Ivanovski et al. [18] said that mixing sodium hypochlorite and dental stone changed the physical properties of the set cast, while Abass and Ibrahim [19] thought that adding calcium hypochlorite to dental stone greatly decreased the material's compressive strength. The extraordinarily infectious nature of COVID-19 (SARS-CoV-2) is a serious cause for concern for the world's population. The fight against this novel virus is being conducted by utilizing a variety of strategies around the world, especially before the vaccination, including the control of its spread. The Environmental Protection Agency of the United States has suggested a number of disinfectants for COVID-19 (HOCl) [20]. The perfect disinfectant and sanitizer would not harm surfaces upon touch, would not corrode, would work well in different formulations, and would not cost too much. HOCl may be considered the disinfectant of choice [21]. Because it is chemical-free, non-toxic, and natural, hypochlorous acid (HOCl) is the most effective chemical against microorganisms without any associated risks [22]. Many researchers investigated the disinfection of dental stone using various disinfectant solutions. To our knowledge, this is the first research that investigates the efficacy of HOCl disinfectant solution on some properties of type III dental stone, such as the reproduction of details and surface hardness [23]. Sodium hypochlorite disinfectant solution was also used in this study because it is one of the ADA-recommended disinfectants in 1:10 dilution to compare the effect of HOCl with that of NaOCl.

Reproduction of details

For the detail reproduction test, the American National Standards Institute (ANSI)/ADA specification number 25 for gypsum products was used. Both the immersion and spray methods of disinfecting type III dental stone samples with a 200 ppm HOCl disinfectant solution did not affect the quality of detail reproduction compared to the control group. This means that all samples in both groups passed the test's requirement of reproducing a groove 0.050 mm wide. These results were in agreement with Abdullah's hypothesis that it is possible to repeatedly immerse and dry a stone cast in the laboratory without compromising the quality of the cast's surface features [22]. Also, this finding is in agreement with a study [24] that found that the stone samples treated with NaOCl and HOCl disinfection solutions retained all of their original surface characteristics after being submerged or sprayed. They looked like the pre-disinfection stone samples, which also had less scores. Such a finding may be due to the accurate and careful manipulation involving the incremental adding of dental stone mixture into the rubber ring mold while being vibrated in order to minimize the entrapment of air bubbles and to ensure the highest possibility of reproducing the grooved surface accurately, especially the 0.050 mm wide groove, regardless of the effect and the method of disinfection with HOCl.

Surface hardness

Dental stone is essential in the fabrication of indirect dental prostheses. The dental stone cast should be hard enough to tolerate the fabrication process [25]. Our investigation showed that the disinfection of type III dental stone samples with HOCl disinfectant solution by both immersion and spray methods caused a significant reduction in their surface hardness when tested after 24 hours of mixing when compared with the surface hardness of the control group. However, a comparison between the disinfection of dental stone samples (type III) by immersion in HOCl disinfectant solution and the disinfection of the same samples by immersion in NaOCl disinfectant solution showed that the reduction in the surface hardness of type III dental stone was less when immersed in HOCl disinfectant solution than when immersed in NaOCl disinfectant solution. In other words, both HOCl and NaOCl disinfectant solutions by immersion method reduce the surface hardness of type III dental stone significantly, but HOCl is better than NaOCl because the reduction in the surface hardness with HOCl was less than that with NaOCl.

Also, our findings showed that there was no significant difference between HOCl and NaOCl by the spray method. The results of the current research cannot be compared to those of earlier studies since HOCl disinfecting solution has never been utilized with dental stone before. This study's results are consistent with those of another [26] that found that the mean hardness of gypsum specimens disinfected with sodium hypochlorite and glutaraldehyde by the immersion technique was lower than that of slurry (control). We found results that agreed with those of Talluri et al. [27], despite their use of a different disinfectant solution, and with those of Moslehifard et al. [28], despite their use of a different measuring method, both of whom found that the surface hardness of dental stones decreased after spraying them with the disinfectants under study. Finally, our results were also in agreement with those of Moslehifard et al. [28] and Vandewalle et al. [29], who found that stone casts immersed in 0.525% sodium hypochlorite showed a reduction in hardness compared to stone casts in slurry and 1% peroxygenic acid. However, the findings of this research contradict those of another study [24], which found that the disinfecting solution increased the surface hardness of most forms of dental stone. It is possible that an interaction between the disinfectant and the stone is to blame for the hardness reduction of gypsum specimens submerged in disinfectant solutions. Even after many washes with running water, a thin coating of disinfectant could still be on the surface of the specimens, causing possible gypsum hardness reduction due to residual disinfectant. The stone's softness was found to be due to the interaction between the disinfectant and the stone, which caused micropores to form [28].

## Conclusions

Type III dental stone can be disinfected using immersion and spray methods without affecting the stone's reproduction of details. From a surface hardness point of view, HOCl disinfectant solution caused a reduction in the surface hardness of type III dental stone by both immersion and spray methods, with less effect by the spray method. However, the effect of the reduction of the surface hardness of type III dental stone after immersion in HOCl was less than that after immersion in NaOCl, while there was no difference between HOCl and NaOCl for the spray method. According to our findings and within the limitations of this study, hypochlorous acid disinfectant was a good choice for cast disinfection and also contamination control for dentists and dental laboratory personnel, but more investigations are needed to evaluate the effect of HOCl disinfectant solution on other properties of type III dental stone such as compressive strength and surface roughness.
